# Acute kidney injury in ECMO patients

**DOI:** 10.1186/s13054-021-03676-5

**Published:** 2021-08-31

**Authors:** Marlies Ostermann, Nuttha Lumlertgul

**Affiliations:** 1 Department of Critical Care, King’s College London, Guy’s and St Thomas’ NHS Foundation Trust, London, UK; 2 Division of Nephrology and Excellence Centre for Critical Care Nephrology, King Chulalongkorn Memorial Hospital, Bangkok, Thailand; 3 Critical Care Nephrology Research Unit, Chulalongkorn University, Bangkok, Thailand

## Abstract

This article is one of ten reviews selected from the Annual Update in Intensive Care and Emergency Medicine 2021. Other selected articles can be found online at https://www.biomedcentral.com/collections/annualupdate2021. Further information about the Annual Update in Intensive Care and Emergency Medicine is available from https://link.springer.com/bookseries/8901.

## Introduction

Extracorporeal membrane oxygenation (ECMO) is a life-saving therapy for patients with severe respiratory and/or cardiovascular failure. There are two main configurations: (1) veno-arterial ECMO (VA-ECMO) in patients with refractory cardiogenic shock or combined cardiorespiratory failure, and (2) veno-venous ECMO (VV-ECMO) in patients with potentially reversible causes of respiratory failure. Over the past decade, use of ECMO has increased substantially in critical care units, emergency departments, interhospital transfers, operating rooms, and during cardiopulmonary resuscitation (CPR) [1].

The in-hospital mortality ranges from 21 to 37% in patients receiving VV-ECMO compared to 40–60% in patients treated with VA-ECMO [2–4]. Despite improving survival in recent years, adverse effects are common including acute kidney injury (AKI), infection, thrombosis, and bleeding [5]. AKI is a frequent complication among patients treated with ECMO, resulting in increased morbidity and mortality [6]. Understanding the impact of AKI, its contributing factors, and renal replacement therapy (RRT) is essential to inform clinical practice and design future studies for prevention and management of this high-risk group.

## Incidence of AKI in ECMO

The reported incidence of AKI in patients treated with ECMO varies from 26 to 85% due to differences in patient characteristics, AKI definition, and clinical settings. The pooled estimated incidence of severe AKI requiring RRT is 45% [6]. AKI is more common in VA-ECMO than in VV-ECMO (61% vs. 46%) and is most often present on the day of ECMO cannulation [6, 7]. The Extracorporeal Membrane Oxygenation for Severe Acute Respiratory Distress Syndrome (EOLIA) and Conventional Ventilatory Support versus Extracorporeal Membrane Oxygenation for Severe Adult Respiratory Failure (CESAR) trials demonstrated a lower incidence of AKI and use of RRT in patients receiving VV-ECMO compared with standard treatment [2].

## Pathophysiology of AKI in ECMO

The underlying mechanisms of AKI in patients treated with ECMO are complex and multifactorial (Table 1).

**Table 1:** Risk factors for acute kidney injury (AKI) during extracorporeal membrane oxygenation (ECMO)

Factors	Pathophysiological mechanisms
Patient-related variables	Hypoperfusion Loss of autoregulation Hypoxia Hypercapnia Nephrotoxins Systemic inflammation Cardiorenal syndrome Increased intrathoracic pressure Increased intra-abdominal pressure Neuro-hormonal effects
IMV-related factors	Biotrauma
ECMO-related factors	PEEP
Hemodynamic variables	Continuous flow (VA-ECMO) Ischemia–reperfusion injury
Hormonal variables	RAAS dysregulation ANP downregulation
Circuit-related factors	Blood shear stress Rhabdomyolysis Hemolysis and oxidative stress Embolism Aortic dissection
Systemic inflammation	Systemic inflammation Renal macro/microcirculatory dysfunction Bioincompatibility Blood/air/surface interaction Hypercoagulable state

*IMV* invasive mechanical ventilation, *PEEP* positive end-expiratory pressure, *VA* veno-arterial, *RAAS* renin–angiotensin–aldosterone system, *ANP* atrial natriuretic peptide

### Patient factors and critical illness

Prior to ECMO initiation, hemodynamic instability, low cardiac output, high intra-thoracic pressure, exposure to nephrotoxic agents, severe hypoxemia, hypercapnia, systemic inflammation/immune-mediated effects, and neurohormonal dysregulation can contribute to AKI [8]. In patients with heart failure, cardiac dysfunction, increased intra-abdominal pressure (IAP), and renal congestion contribute to impaired renal blood flow and cardiorenal syndrome [9]. AKI might also occur in the context of other critical illness-related complications including bleeding, limb ischemia, infection, and coagulopathy [8].

### Impact of mechanical ventilation

Invasive mechanical ventilation is associated with altered hemodynamics and release of pro-inflammatory cytokines (e.g., tumor necrosis factor [TNF]-α, inter-leukin [IL]-1β, IL-6 and IL-8) [10]. Plasma cytokine concentrations are predictive of AKI development and renal non-recovery [11]. The application of positive end-expiratory pressure (PEEP) has several beneficial effects for lung recruitment and decrease in left ventricular (LV) pre- and afterload. However, increasing PEEP and/ or tidal volumes may elevate intrathoracic pressure, reduce venous return, decrease cardiac output, and increase right ventricular (RV) afterload, resulting in elevated systemic venous pressure, venous congestion, and reduction of renal perfusion. In addition, fluid retention may develop as a result of activation of the sympathetic nervous system (SNS) and renin–angiotensin–aldosterone system (RAAS), and suppression of atrial natriuretic peptide (ANP) release [9]. Lung-protective ventilation limits lung injury and has potential to reduce the risk of AKI [12]. However, permissive hypoxia and hypercapnia might ensue and decrease renal blood flow [9].

### ECMO-related factors

Following ECMO cannulation, an improvement in oxygenation helps restore the microcirculation in previously hypoxic and hypoperfused organs and tissues, often in association with a degree of ischemia–reperfusion injury and production of reactive oxygen species (ROS) [13]. Continuous flow during VA-ECMO reduces pulsatility, which may compromise renal cortical blood flow and upregulate the RAAS inducing systemic vasoconstriction [14]. Circuit-related factors contributing to the development of AKI include hemolysis, rhabdomyolysis from local ischemia, hemorrhage, renal microthrombosis, and cannula-related complications (e.g., malposition of the cannula leading to venous obstruction, cholesterol embolism following cannulation, aortic dissection) [15, 16]. Hemolysis may occur due to a combination of shear stress from blood travelling through the blood pump, negative intra-circuit pressures, and contact with the non-biological and non-endothelialized surface of ECMO membranes [15]. This leads to elevated plasma free hemoglobin, release of free iron, oxidative stress, and filtered heme pigments causing tubular obstruction [17]. Blood exposure to artificial surfaces also induces the release of inflammatory cytokines, complement and leukocyte activation, and hypercoagulability. Finally, 74 although VA-ECMO improves oxygenation and peripheral circulation, limited LV off-loading combined with low ejection fraction can result in LV overdistension and worsening pulmonary edema.

## Risk factors for AKI

Reported risk factors for AKI during ECMO are older age, pre-existing comorbidities (e.g., cirrhosis), post cardiotomy shock as indication for ECMO, late implantation of ECMO, reduced LV ejection fraction (LVEF), intraoperative transfusion, high lactate, high plasma free hemoglobin, increased bilirubin, and high neutrophil to lymphocyte ratio [18]. Red blood cell distribution width >14.1%, a marker of inflammation and anemia, has also been found to be associated with an increased risk of severe AKI [19]. During ECMO, high inotropic equivalents, ECMO pump speed, and ECMO duration are linked to AKI development [20]. Higher pump speeds are associated with hemolysis, leukocyte and platelet destruction, and complement activation [21]. To prevent heme pigment-associated AKI, pump revolutions/min (RPM) should be limited to safe levels to avoid excessive negative pressures. AKI patients who required RRT whilst receiving ECMO were more likely to be treated with VA-ECMO, had more organ dysfunction at the time of ECMO insertion, and required more transfusions [22].

## RRT and ECMO

### Indications

Fluid overload is highly prevalent and associated with higher mortality and pro-longed ECMO duration [23]. According to a recent survey, fluid overload management (43%) or prevention (16%) are the predominant triggers for RRT initiation during ECMO, followed by AKI (35%), and electrolyte disturbances (4%) [24].

### Timing

Theoretically, early initiation of RRT may help resolve fluid overload faster and achieve better sodium removal per unit volume than diuretics in ECMO patients. In general ICU patients, recent randomized controlled trials (RCTs) not only failed to demonstrate the survival benefits of early over standard initiation strategy, but also showed increased harm in the early-initiation group including an increased risk of dialysis dependence at 90 days and adverse events [25–27]. A post-hoc sub-analysis of The Artificial Kidney Initiation in Kidney Injury (AKIKI) trial in acute respiratory distress syndrome (ARDS) also demonstrated similar outcomes between early and standard initiation strategies [28]. Another study using propensity-score matching compared early versus late initiation of CRRT after ECMO (median time from ECMO to CRRT initiation 1 vs. 15 days) and found no difference in survival [29].

In light of the fact that serum creatinine, AKI stage and urine output are poor markers to guide initiation of RRT, the demand-capacity concept has been proposed as a method to guide the decision-making process. Accordingly, RRT should be considered if the degree of fluid overload and AKI-related metabolic derangements are likely to overwhelm the kidneys’ capacity to compensate, and pharmacological measures (diuretic therapy, sodium bicarbonate) are unlikely to be effective [30]. The expert committee of the 21st Acute Dialysis Quality Initiative (ADQI) meeting concluded that there was no evidence of benefit for pre-emptive use of RRT in patients treated with ECMO [9]. Therefore, the decision to initiate RRT in patients receiving ECMO should be based on usual absolute and relative indications for critically ill patients.

### Modality

RRT options include continuous RRT (CRRT), prolonged intermittent renal replacement therapy (PIRRT), intermittent hemodialysis (IHD), and peritoneal dialysis. Each modality has advantages and disadvantages (Table 2). CRRT and peritoneal dialysis are suitable for patients with hemodynamic instability although CRRT enables more precise fluid and electrolyte management. Meanwhile, PIRRT and IHD allow planned circuit downtime. It is possible to provide CRRT, PIRRT and IHD via integration into the ECMO circuit or separately. When choosing CRRT, any mode of clearance can be delivered, namely slow continuous ultra-filtration (SCUF), continuous veno-venous hemofiltration (CVVH), continuous veno-venous hemodialysis (CVVHD), and continuous veno-venous hemodiafiltration (CVVHDF).

**Table 2:** Advantages and disadvantages of each renal replacement modality during extracorpo-real membrane oxygenation (ECMO)

Modality	Advantages	Disadvantages
IHD	Integration in ECMO circuit possible Reduced filter downtime Lower costs than CRRT	Need for more rapid fluid removal Risk of hemodynamic instability Disequilibrium syndrome
PIRRT	Integration in ECMO circuit possible Reduced filter downtime Lower costs than CRRT Slower volume and solute removal than IHD	Risk of hemodynamic instability in high-risk patients
CRRT	Integration in ECMO circuit possible Continuous fluid and solute removal Allows more precise control of fluid balance Better hemodynamic stability	Patient immobilization Increased risk of hypothermia High costs
PD	Better hemodynamic stability Technically simple Lower cost No addition of anticoagulation	Less experience in adult patients Requires specific intraperitoneal catheters Risk of peritonitis Risk of hyperglycemia May interfere with diaphragmatic movements

*IHD* intermittent hemodialysis,* PIRRT* prolonged intmeromviettmenetn thsemodialysis,* CRRT* continuous renal replacement therapy,* PD* peritoneal dialysis

### Techniques

There are three ways to provide RRT with ECMO: using an in-line hemofilter, connecting a RRT device to the ECMO circuit (integrated system), or using a separate RRT access from the ECMO circuit (parallel system) [31, 32] (Table 3). In the absence of evidence-based data, practice is based on expert opinion, availability of machines, local expertise, and staff organization. A 2013 survey of 65 ECMO centers showed that 50.8% of centers used independent CRRT circuits while 21.5% used in-line hemofilter [24]. The results of a recent survey of ECMO centers in France and Switzerland are awaited.

**Table 3:** Advantages and disadvantages of RRT techniques during extracorporeal membrane oxygenation (ECMO) [31, 33]

Techniques	Advantages	Disadvantages
In-line hemofilter	Low cost Generates large volumes of UF No need for separate anticoagulation Small priming volume	No pressure monitoring Requires external infusion device to control UF and deliver replacement fluid Less precise UF Limited solute clearance Flow turbulence and risk of hemolysis
Independent RRT access (parallel system)	Allows fine-tune adjustment of solute and fluid removal Able to provide RRT independent of ECMO Allows use of regional anticoagulation Simplified circuit changing without need for perfusionist Mode of solute clearance not restricted	Need for separate vascular access Risk of mechanical and infectious complications Higher extracorporeal blood volume Technically more complex to manage two separate circuits
RRT connected to ECMO circuit (integrated system)	Allows fine-tune adjustment of solute and fluid removal Mode of solute clearance not restricted No need for separate vascular access Avoids complications related to line insertion	Pressure alarms (low pressure alarms if connected pre-pump and high pressure alarms when connected post-pump) Requires a RRT machine capable of adjusting alarm settings Risk of air entrapment if access line is connected before centrifugal pump Flow turbulence with risk of hemolysis and thrombus formation Generation of shunt within ECMO circuit Recirculation

*RRT* renal replacement therapy, *UF* ultrafiltration

#### In-line hemofilter

**Fig. 1:**
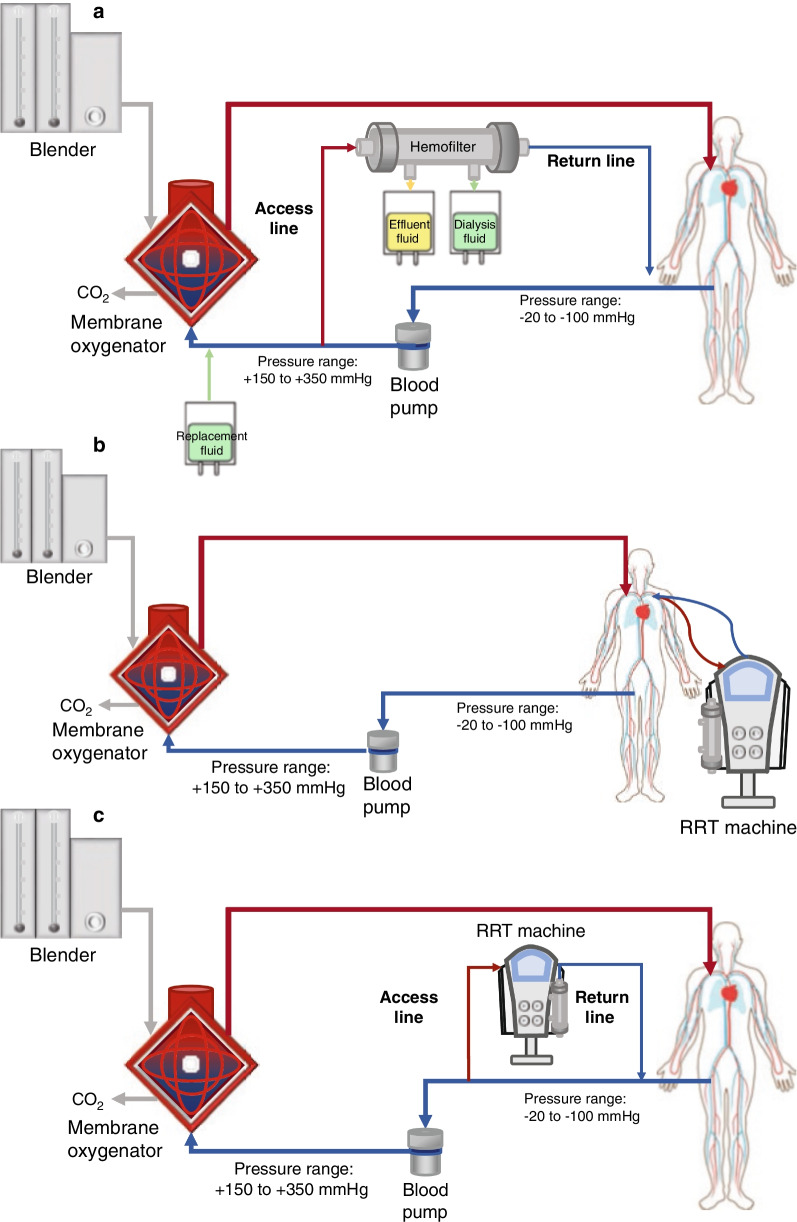

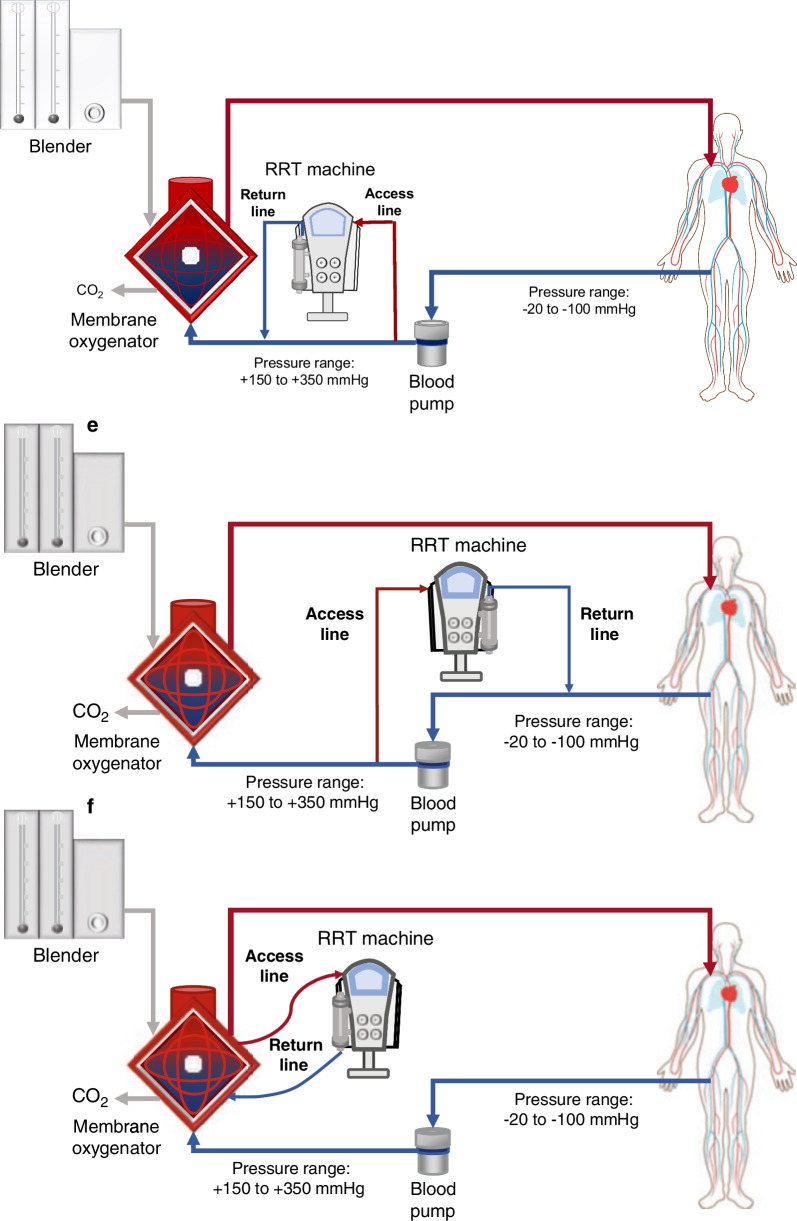
Options of combining extracorporeal membrane oxygenation (ECMO) and continuous renal replacement therapy (CRRT) circuits. **a** An in-line hemofilter is integrated into the ECMO circuit. Replacement fluid is directly administered into the ECMO circuit. Alternatively, dialysis fluid can be supplied in a counter-current position. Replacement/dialysis fluid rates and ultrafiltration rates can be controlled via infusion pumps. **b** The CRRT device is connected to the patient through a separate catheter independent of the ECMO circuit. **c** The access (inlet) and the return (outlet) lines of the CRRT device are connected before the centrifugal blood pump (low-pressure part) of the ECMO circuit. **d** Both the access and the return lines of the CRRT device are connected after the blood pump. **e** The access line of the CRRT device is connected after the blood pump (high-pressure), while the CRRT return line is connected before the centrifugal blood pump. **f** The access line of the CRRT device is connected directly after the membrane oxygenator, while the return line is connected directly before the oxygenator

It is possible to provide RRT by incorporating a hemofilter into the ECMO circuit [9, 15, 31] (Fig. 1a). The hemofilter is placed after the pump pre-oxygenator so that the oxygenator can trap air and clots. The positive pressure from the ECMO circuit will forward the blood flow through the hemofilter. Then, blood is returned from the filter to the ECMO circuit before the pump. The blood flow rate in the hemofilter is the difference between the total ECMO blood flow rate and the actual flow delivered to the patient, which is measured by placing an ultrasonic probe on the arterial return line from the ECMO circuit. This technique is mainly used for ultrafiltration via the SCUF mode. CVVH or CVVHD can be delivered by adding replacement fluid (CVVH) or dialysis fluid (CVVHD) through standard infusion pumps. Ultrafiltration rate is regulated by connecting a standard infusion device to the effluent port of the hemofilter. However, the amount of removed fluid is less accurate and prone to error up to 800 ml/day [34]. A more precise method is to weigh the actual volume of ultrafiltration using a scale or a volumetric measuring device but this method is labor-intensive. Since hemofilters are not designed for use with high pressure systems and the maximal volume of the infusion pump is limited at 1 l/h, convective and diffusive clearance are less effective than with CRRT using conventional membranes. The hemofilter blood flow rate can be adjusted via a stopcock or a flow-restrictor. Nevertheless, the generated turbulent flow might cause hemolysis and trigger thrombus formation. Most importantly, there is no pressure monitoring with this technique, which may lead to delayed detection of hemolysis, filter rupture or clot formation.

#### Parallel system (independent RRT access)

Setting up an independent RRT device is simple as it does not require ECMO circuit manipulation (Fig. 1b). Dose and modality can be adjusted, and fluid balance can be controlled in a precise manner as per usual protocol. Regional anticoagulation can also be added and optimised to prolong filter longevity. However, the need to insert a separate vascular access whilst systemically anticoagulated poses a risk for line-related mechanical and infectious complications. This technique also requires an additional extracorporeal blood volume, which might interfere with ECMO performance [9, 15, 31].

#### Integrated system (combining RRT machine into the ECMO circuit)

There are several ways to incorporate a RRT device into the ECMO circuit using two high-flow Luer lock three-way taps as connectors to the RRT access (inlet) line and return (outlet) line [9, 31, 33, 35] (Fig. 1c–f). The pressure before the ECMO pump is negative (range: − 20 to − 100 mmHg) and post-pump is positive (range: + 150 to + 350 mmHg), which might interfere with the RRT circuit. If a centrifugal pump is used, the RRT access line should be placed post-pump (either before or after the oxygenator) to avoid air entrapment (Fig. 1d, e). The RRT return line should be connected before the oxygenator (either pre- or post-pump) to avoid air embolism, clot formation and venous admixture. Careful attention should be paid to the inherently set pressure limits of the ECMO circuit and RRT devices from different manufacturers. The default access pressure of the RRT machine is typically negative. High post-pump pressure may trigger pressure alarms at the entry point of the RRT machine although current RRT machines can tolerate higher pressures or allow adjustment of alarm settings up to + 350 to + 500 mmHg. To overcome the alarm limits of the RRT machine, a long monitoring extension line attached to the RRT access tubing or reducing the blood flow rates can help to lower the pressure from the ECMO circuit [8]. Another option is to use clamps on the connectors or flow restrictors placed outside the tubing to adjust the RRT circuit pressure on the access or, alternatively, on the return line to avoid extreme pressures [36]. However, this may cause turbulence in blood flow and trigger hemolysis or thrombosis. When withdrawing blood post-oxygenator and returning blood before pump, re-circulation in the RRT circuit (shunt within a shunt) and RRT underdosing may occur. Alternatively, the access and return lines may be safely connected through Luer locks immediately before and after the oxygenator to avoid pressure alarms (Fig. 1f).

Combining RRT into the ECMO circuit has several benefits. No additional vascular access is needed and complications related to line insertion are avoided. This method is more effective than using an in-line hemofilter and provides more precise ultrafiltration control and solute clearance by any modality of choice. Use of the heater on the CRRT device is optional. There is also no need for routine additional anticoagulation. However, every connection and disconnection requires support from an ECMO specialist/perfusionist and might pose a risk of air embolism/clot to both devices.

#### Comparison of different techniques

There are few studies comparing the efficacy between the different techniques. Compared with an in-line hemofilter technique, integrating a RRT machine into the ECMO circuit was shown to provide more accurate fluid management [37]. A recent study concluded that independent RRT access was associated with fewer effective sessions and shorter filter lives in comparison with the integrated system [38]. However, the average prescribed RRT dose was 40 ml/kg/h, which is higher than currently recommended. In addition, the CVVH modality was used with 33% of replacement fluid given pre-filter, which might result in a relatively high filtration fraction. Regional citrate anticoagulation was not used [39]. Another study also reported longer filter life for the integrated system compared with the parallel technique [35]. With regards to clearance, a recent study demonstrated similar efficacy for solute clearance and ultrafiltration between the parallel and the integrated methods [40].

### Technical aspects

#### Mediator removal

Although raised cytokine concentrations have been demonstrated in patients with AKI receiving ECMO and might be implicated in multiorgan dysfunction [41], there is insufficient evidence to recommend blood purification therapy outside the setting of AKI for patients receiving ECMO. Therefore, the use of RRT and/or hemoadsorption with the sole intention of clearing pro- or anti-inflammatory mediators during ECMO is not recommended [9].

#### Anticoagulation

Systemic infusion of unfractionated heparin is the standard anticoagulation in patients receiving RRT and ECMO unless contraindicated. However, significant clotting or excessive bleeding precluding the use of systemic heparin may require the addition of regional citrate anticoagulation to ensure effective RRT delivery [42]. Nevertheless, significant citrate dilution might occur. If the RRT access line is connected from the post-oxygenator limb and the return line is connected to the pre-oxygenator limb, infused citrate will be partially delivered and mixed with pre-oxygenator blood. This may reduce clotting in the oxygenator. Calcium should be infused via a separate central venous access to reduce clotting in the system.

#### Drug dosing

ECMO and RRT can significantly alter the pharmacokinetics of medications such as antibiotics and sedatives, yet little is known about the optimal regimen for patients treated with both RRT and ECMO. Generally, ECMO increases the volume of distribution and reduces drug clearance. The ECMO circuit might act as a reservoir and redistribute the sequestered drug back into the patient leading to prolonged effects, especially of lipophilic medications with a large volume of distribution (e.g., voriconazole, propofol, fentanyl, midazolam) [8]. In contrast, the ECMO membrane and tubing may adsorb some drugs and reduce plasma concentrations. The presence of RRT increases the risk of both under- and over-dosing further. A preliminary analysis showed that standard dosing of meropenem (1 g 8-hourly) is likely to maintain sufficient trough concentrations (> 2 mg/l) to treat highly susceptible gram-negative pathogens but might be inadequate for higher trough targets [43]. Individualizing drug regimens in patients receiving concomitant ECMO and RRT using therapeutic drug monitoring is suggested where possible until more pharmacokinetic data become available.

## Short-term outcomes

AKI and RRT have been shown to be independently associated with mortality but it is uncertain whether they directly increase the risk of dying or merely represent the acuity and severity of the illness [19]. The pooled estimated hospital and/or 90-day mortality rates of patients with AKI and severe AKI requiring RRT while on ECMO were 62.0% and 68.4%, respectively [6]. The likelihood of dying in hospital of ECMO patients receiving RRT is three times that of those without RRT [6]. Importantly, mortality has decreased by > 20% since 2016 compared with data from before 2015, possibly due to better patient selection, timing, and clinical application [44].

AKI requiring RRT is associated with other complications, including sepsis, need for fasciotomy/amputation, respiratory failure, intra-aortic balloon pump (IABP) usage, massive blood transfusion, and failure to wean from ECMO [45]. Acute respiratory failure can also be worsened following AKI due to fluid overload, pulmonary edema, increased inflammatory mediators and increased risk of intercurrent sepsis. Other risk factors for mortality include age, oliguria, AKI stage 3, RRT duration hypercapnia, high sequential organ failure assessment (SOFA) score, blood loss, transfusion requirement, hemodynamic instability, liver failure, low Glasgow coma score, and fluid overload [7, 46, 47].

## Renal recovery and long-term outcomes

The long-term renal prognosis in ECMO survivors is uncertain. Previous studies showed high rates of liberation from dialysis at hospital discharge [4, 48]. However, only 42% of AKI stage 3 survivors had complete renal recovery [7]. In a cohort of post-cardiotomy patients with cardiogenic shock receiving VA-ECMO, all but 2 patients recovered from AKI stage 3 at 6 months [18]. However, it should be noted that creatinine at discharge might be falsely low due to loss of muscle mass and malnutrition following prolonged hospitalization. Therefore, low serum creatinine levels may lead to erroneous glomerular filtration rate (GFR) results and mislead clinicians, resulting in inappropriate drug dosing and inadequate follow-up.

It is established that AKI survivors are at increased risk for long-term mortality, end-stage kidney disease, chronic kidney disease (CKD), and poorer quality of life. However, only a few studies have explored the long-term outcomes of ECMO patients with AKI. In children, two large ECMO studies independently reported a 20-year experience and showed no incidence of end-stage kidney disease in the absence of primary renal disease [38, 48]. In contrast, analysis of a VA-ECMO cohort of adult population showed an 85% incidence of major adverse kidney events, comprised death, end-stage kidney disease, and reduced GFR at 1 year [49].

Risk factors for 1-year major adverse kidney events included lower GFR at baseline, higher AKI stage at ECMO cannulation, and number of red blood cell transfusions. Moreover, the median GFR decline was 20 ml/min/1.73 m^2^, and half of AKI survivors had a GFR decline of more than 30%. Decline of GFR by > 30% is associated with > 5 times increased risk of end-stage kidney disease [50]. Therefore, the risk of serious long-term renal outcomes should not be underestimated in ECMO patients with AKI. Analysis of a national Taiwan database including 3200 adult patients receiving ECMO with up to 10-year follow-up data revealed higher rates of all- cause mortality, end-stage kidney disease and CKD in patients with RRT-requiring AKI compared with non-dialysis-requiring AKI patients [45]. Prolonged CRRT use (> 7 days vs. ≤ 6 days) was associated with an increased risk of end-stage kidney disease, ventilator dependence, and readmission rate but not survival after discharge [51].

## Conclusion

AKI is extremely common and associated with worse short-term and long-term outcomes in patients receiving ECMO, especially when RRT is required. The most common indication for RRT initiation in these patients is fluid control. RRT should be initiated when the anticipated demand from fluid overload and metabolic derangements exceeds the capacity of the kidneys to compensate. The modality and techniques of providing RRT in patients receiving ECMO depend on local practice and expertise. Provision of RRT as an armamentarium of multiorgan support therapy requires a multidisciplinary team engagement (such as intensivists, nephrologists, cardiologists, cardiac, thoracic and vascular surgeons, perfusionists, dedicated nurses, pharmacists, dietitians, and others) from admission, through ECMO cannulation and RRT initiation, until after discharge. Further research should determine the optimal technique to combine ECMO and RRT, optimal drug dosing and long-term renal prognosis.

## Data Availability

Material used in this manuscript is available from the corresponding author at reasonable request.
